# Is tuberculosis patients management improved in the integrated TB control model in West China? A survey in Guizhou Province, China

**DOI:** 10.1186/s40249-019-0563-3

**Published:** 2019-07-02

**Authors:** Jie Pu, Wei Chen, Wei-Xi Jiang, Wei Xing, Sheng-Xiang Liang, Geng Wang, Shi-Li Liu, Hao Wu, Ying Li, Sheng-Lan Tang

**Affiliations:** 1 Department of Social Medicine and Health Service Management, Army Medical University (Third Military Medical University), No. 30 Gaotanyan Road, Shapingba District, Chongqing, 400038 China; 2 Department of TB Control, Center of Disease Control and Prevention, Guiyang, 550004 Guizhou Province China; 3 Duke Kunshan University, Kunshan, 215316 China; 4 Second Affiliated Hospital, Army Medical University (Third Military Medical University), No. 83 Xinqiao Road, Shapingba District, Chongqing, 400037 China; 5 Duke Global Health Institute, Duke University, Durham, NC 27708-0065 USA

**Keywords:** Tuberculosis, Community-based, Supervised treatment, Patient-centered treatment, TB patient management

## Abstract

**Background:**

Tuberculosis (TB) patient management (TPM) is crucial to improve patient compliance to treatment. The coverage of TPM delivered by TB dispensaries or Centers for Disease Control and Prevention (CDC) was not high under the previous CDC model of TB control in China. In the integrated TB control model in China, TB patient management (TPM) was mainly delivered by lay health workers (LHWs) in primary health care (PHC) sectors. This study aims to investigate TPM delivery in resource-limited western China and to identify factors affecting TPM delivery by LHWs under the integrated TB control model.

**Methods:**

A stratified random sampling was used to select study sites. Pulmonary TB (PTB) patients ≥15 years old from selected counties/districts in Guizhou Province were surveyed from August 2015 to May 2016. Structured questionnaires were used to collect data. A *χ*^2^ test and logistic regression were used to identify factors associated with self-administered treatment (non-TPM).

**Results:**

In total, 638 PTB patients were included in the final analysis. Close to 30% of patients were ethnic minorities. More than 30% of patients were from counties with high TB burden, and 24.9% of patients had poor compliance to treatment. Only 37.1% of patients received TPM delivered by LHWs under the integrated TB control model throughout the treatment period. The main reasons for unwillingness to manage reported by patients included social stigma and no perceived need. Being ethnic minorities (*OR* = 3.35) was a main factor associated with lower likelihood of receiving TPM, while living in areas with middle or high TB burden may increase the likelihood of receiving TPM (*OR* = 0.17 and 0.25, respectively). Among current management approaches, more than 85% of patients chose phone reminder as their preferred TPM by LHWs.

**Conclusions:**

TPM under the integrated model in West China is still low and need further improvement, and the impeding factors of TPM need to be addressed. Strengthening patient-centered and community-based TPM and developing more feasible approaches of TPM delivery should be explored in future research in this region.

**Electronic supplementary material:**

The online version of this article (10.1186/s40249-019-0563-3) contains supplementary material, which is available to authorized users.

## Multilingual abstracts

Please see Additional file 1 for translations of the abstract into the five official working languages of the United Nations.

## Background

A World Health Organization (WHO) report in 2018 indicated that tuberculosis (TB) is still one of the top 10 causes of death worldwide, and is the leading cause of death from a single infectious agent (above HIV/AIDS) [1]. Globally, 10 million people developed TB disease in 2017 [1]. China has the second highest TB burden and multidrug-resistant TB (MDR-TB) burden in 2017 [1].

Patient compliance to anti-tuberculosis treatment is the key to healing and avoiding drug resistance [2]. However, a significant proportion of TB patients often experience treatment interruption and default before completion of treatment in many countries [3]. Therefore, TB patients should be managed and monitored during treatment to ensure treatment compliance and allow for the identification and management of adverse drug reactions [2]. The WHO recommended directly observed treatment (DOT) in the 1990s, which improves TB patient management (TPM) and compliance [4]. However, many studies have reported that DOT is not always implemented for all PTB patients in the “Centers for Disease Control and Prevention (CDC) model” [5, 6].

Since the implementation of the 12th Five year plan of the National TB Program in 2011, China’s TB control model transformed from the “CDC model” to the “integrated model” in most regions [7]. In the CDC model, the CDC/TB dispensary provides both clinical treatment and TPM, and the primary health care (PHC) sectors (including community health centers, township health centers or village clinics) are only required to refer suspected TB cases to the CDC [7]. In the integrated model, the clinical treatment is provided by designated medical institutions, mostly comprehensive hospitals. The provisions of TPM, including patient referral, defaulter tracing, and providing treatment supervisions for the TB patient and community TB health education, transfer to the PHC sectors while the TB dispensary/CDC is mainly responsible for general public health care [8]. TPM by lay health workers (LHWs) in PHC sectors is emphasized more in an integrated model.

Many studies have evaluated TPM in a CDC model and demonstrated that TPM needs improvement [5, 6], especially for the resource-limited West China. One study [9] reported that only 50% of TB patients were managed by LHWs in PHC sectors in West China. Previous research also investigated social factors associated with implementation of TPM under the CDC model and found that rural patients are more likely to self-administer treatment mainly due to poverty, difficulty in mobility, being busy with work, low education level and lack of health consciousness [6, 10, 11]. Studies also revealed that TB patients often reject DOT by LHWs through home visits because of TB-related stigma [6, 10, 12, 13] and patients perceived no need for supervision regarding anti-TB drug intake [6, 10, 12]. For TPM management in the integrated model, studies in more developed regions in China reported that comprehensive TPM coverage was improved [14–19]. However, in West China, studies only evaluated TPM in terms of patient referral [20] and tracing rate, and few reported comprehensive TPM in the integrated model in resource-limited and mountain areas with high TB burden. Moreover, it remains unclear whether impeding factors of TPM under the CDC model still affect TPM under the integrated model in those areas. This study aimed at evaluating TPM status in Guizhou, a province typical of the less developed inland provinces with high TB burden in China [21, 22], and analyzing factors associated with insufficient delivery of TPM and exploring needs of TPM by LHWs in PHC sectors under the integrated model.

## Methods

### Study sites

We conducted a cross-sectional study in Guizhou Province from August 2015 to May 2016. The fifth national TB epidemiological survey in 2010 revealed that the active and smear positive TB prevalence (per 100 000) in Guizhou was 1226 and 231, respectively [10], which is much higher than the national level (459 and 66, respectively) and higher than that in western China (695 and 105, respectively) [9]. The incidence of TB in Guizhou is ranked the third highest in China following that in Xinjiang and Tibet [11], and the prevalence of acquired MDR-TB cases in Guizhou is higher (45.1%) than the national average (25.6%) [12]. TB control efforts started late in Guizhou, and the entire province was not covered by the modern TB control strategy until 2005 [10, 13] when the integrated TB control model launched across all counties/districts in Guizhou.

**Fig. 1:**
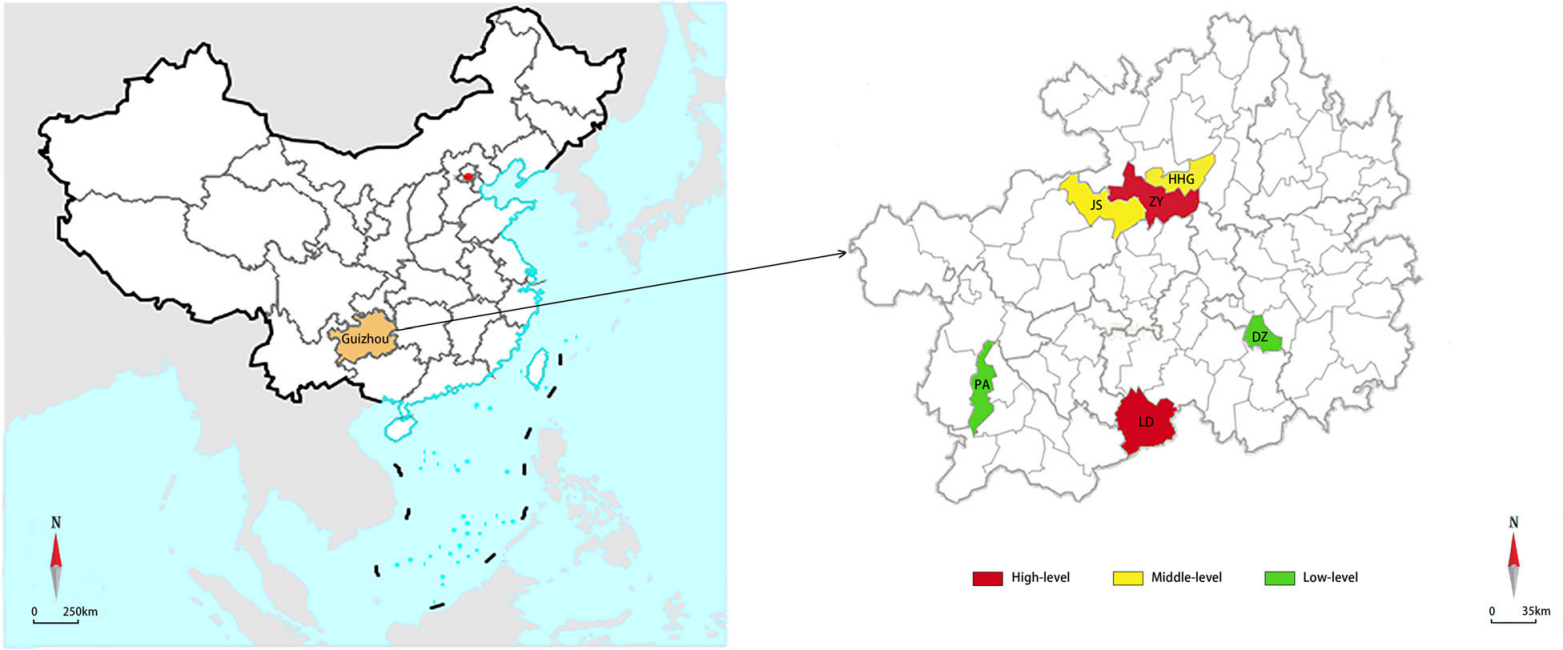
Map of study place in Guizhou, China. This figure described the sampling counties: counties colored with red have a high-level burden of tuberculosis; counties colored with yellow have a middle-level tuberculosis burden; counties colored with green have a low-level tuberculosis burden

A stratified random sampling method was used to select study sites as follows. First, all counties/districts in Guizhou Province were grouped into three levels according to their TB incidence in 2014: the high-level TB burden (TB incidence in the highest 30%), the low-level TB burden (TB incidence in the lowest 30%), and the mid-level TB burden (TB incidence in the remaining 40%). Then, from each group of counties/districts, two counties/districts were randomly selected as study sites. A total of six counties/districts were included in this study (Fig. 1): Luodian (LD) and Zunyi (ZY) with high level TB burden, Jinsha (JS) and Honghuagang (HHG) with middle level TB burden, Puan (PA) and Danzhai (DZ) with low level TB burden. The integrated TB control model was established in all included counties/districts.

### Study participants

All adult pulmonary TB (PTB) patients who met the following criteria were targeted for recruitment in the six selected counties/districts: (1) registered at TB dispensaries and were diagnosed as drug-sensitive PTB according to NTP criteria, (2) newly diagnosed and retreatment PTB patients diagnosed in the past six months and who received anti-TB drug treatment for at least two months, and (3) aged 15 years and older. Patients of ethnic minorities were also included in the survey. Patients with extrapulmonary tuberculosis were excluded.

Patient recruitment was facilitated by TB dispensaries in the study counties/districts. During recruitment, potential participants were provided with a detailed explanation about the study objectives. Those who expressed interest were asked to read the informed consent form and were assured of confidentiality. Those who were willing to participate in the study were then asked to sign the informed consent form. People who could not express themselves clearly (who had disturbance of consciousness or difficulties with speech or hearing) or who were unwilling to participate in the survey were not included.

### Data collection

A structured questionnaire survey was conducted in clinic rooms at the district TB dispensaries or designated hospitals. All questionnaires were administered by trained investigators. Data collected from all counties and districts included the following: (1) questions on sociodemographic profile (age, sex, occupation, ethnicity, education, registered residence, marital status, etc.), (2) TB patient treatment experience, (3) TB patient management status, and (4) TB patients’ satisfaction and needs of community-based management.

### Data analysis

Data were entered using Epi Data 3.0 (The EpiData Association, Odense, Denmark) and were analyzed using the Statistical Package for Social Science (SPSS 19.0, IBM Corporation, Armonk, NY, USA). A two-tailed probability level of *P* < 0.05 was considered statistically significance. Missing data were excluded from the analysis. Numbers and percentages were used to describe the characteristics of study subjects. Descriptive analysis was used to present TPM status and the means and satisfaction of TPM by LHWs. The Chi-square test was used to screen factors associated with patients’ self-administered medication in the intensive phase and the continuation phase of treatment (yes = 1, no = 0). Significant factors based on the Chi-square test (*P* < 0.05) were identified by multivariate logistic regression analysis.

## Results

### Demographic characteristics of PTB patients and their treatment behavior

A total of 671 PTB patients were recruited to participate in the survey, and 24 declined (response rate was 95.1%). Nine patients < 15 years old were excluded, and finally 638 were included in the analysis. Of the patients surveyed, more than 60% (*n* = 423) were 20 to 60 years old, and a majority of them were male patients (61.1%, *n* = 389). Close to 30% (*n* = 185) of patients were ethnic minorities. A high proportion of the patients (*n* = 572, 89.9%) were rural residents. Nearly half (49.1%, *n* = 311) of the patients only had primary school and lower education, and 70.0% (*n* = 448) of the patients were farmers or migrant workers. Almost all of the patients (96.4%, *n* = 613) had access to the national basic medical insurance. A majority (87.8%, *n* = 560) were newly diagnosed PTB patients and approximately 40% (*n* = 256) were smear negative. More than 30% (*n* = 272) PTB patients were from counties with high TB burden. Regarding their treatment behavior, most (81.0%, *n* = 516) of the patients lived close to primary health facilities, but only 15% (*n* = 96) of the patients had their initial consultation in PHC sectors. Regarding compliance with treatment, 24.9% (*n* = 159) of the patients had poor compliance (experienced missing doses of anti-TB drugs, default or irregular follow-up with sputum smear test) (Table 1).

**Table 1:** Demographic characteristics of pulmonary tuberculosis patients in questionnaire survey

Demographic characteristics	Frequency	Percentage
Age (*n* = 638)		
< 20	75	11.8
20–40	229	35.9
40–60	194	30.4
≥ 60	140	21.9
Gender (*n* = 637)		
Male	389	61.1
Female	248	38.9
Ethnicity (*n* = 633)		
Han Race	448	70.8
Others	185	29.2
Residence (*n* = 637)		
Urban	65	10.2
Rural	572	89.8
Registered information (*n* = 632)		
Resident	563	89.1
Migrant	69	10.9
Marital status (*n* = 630)		
Single	154	24.5
Married	426	67.6
Divorced/Widowed	50	7.9
Education (*n* = 633)		
Primary and below	311	49.1
Junior middle school	176	27.8
High school and above	146	23.1
Occupation (*n* = 638)		
Staff/Cadre/Retiree	42	6.6
Self-employed	33	5.2
Farmer/Migrant worker	448	70.2
Student	56	8.8
Others	59	9.2
Main source of income (*n* = 631)		
Patients	108	17.1
Shared with other	278	44.1
Others	245	38.8
Economic status (*n* = 635)		
Labor force	415	65.4
Dependant	220	34.6
Health insurance (*n* = 636)		
Basic health insurance	613	96.4
Others	23	3.6
TB burden (*n* = 638)		
Low	181	28.4
Middle	221	34.6
High	272	37.0
Type of patient (*n* = 638)		
New	560	87.8
Retreatment	78	12.2
AFB smear status (*n* = 638)		
Negative	382	59.9
Positive	256	40.1
First health facility for consultation (*n* = 638)		
Primary health facility	96	15.0
Non-primary health facility	542	85.0
Nearest health institution *(n* = 637)		
Primary health facility	516	81.0
Non-primary health facility	121	19.0
Adherence to treatment (*n* = 637)		
Adherence	478	74.9
Missed dose	92	14.4
Interrupted treatment	40	6.3
No follow-up sputum exam	27	4.2
Willingness to receive TB treatment management (*n* = 637)		
Yes	380	59.7
No	257	40.3

*TB* Tuberculosis, *AFB* Acid-fast bacilli

### Situation of TPM and willingness to receive TPM by LHWs

**Fig. 2:**
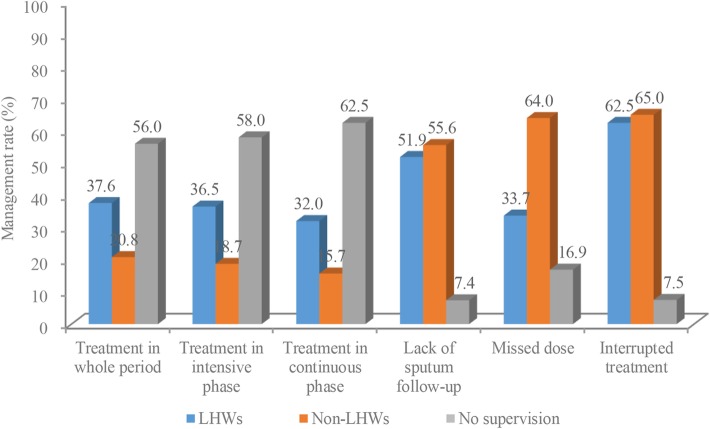
Tuberculosis treatment management reported by tuberculosis patients in Guizhou Province (%). This figure showed the percentage of tuberculosis patient who received management in intensive phase–continuous phase–when they missed dose–interrupted treatment–or lacked of follow-up LHWs: Lay health workers.

Among all of the patients, only 37.1% (237) received TPM by LHWs during the entire period, and the percentage was 36.5% in the intensive phase and 32% in the continuation phase. More than half of the participants had taken medicine by themselves during both the intensive phase and the continuation phase of treatment, and the remaining 5.5% of patients were supervised by family members or a TB doctor in TB dispensaries (Fig. 2). For patients who experienced any form of noncompliance to treatment, 62.5% of those who experienced treatment interruption, 51.9% of those who lacked a regular follow-up sputum exam, and 49.4% of those who missed doses received TPM by LHWs (Fig. 2). Of patients who received TPM by LHWs, most (94.1%) were reminded to take medicine through telephone by LHWs. Similarly, telephone reminders were used often for TPM when patients forgot to take medicine, discontinued treatment and did not go for follow-ups on time (see Additional file 2).

Regarding the willingness to receive TPM by LHWs, approximately 40% of TB patients were unwilling to receive TPM by LHWs in both intensive and continuous treatment phases. Nearly 90% of patients were willing to receive TPM if they missed a dose, interrupted treatment or missed a sputum follow-up. More than 85% of patients chose phone reminder as their preferred TPM. The main reasons for unwillingness to receive management included social stigma and no perceived need (Table 2).

**Table 2:** Patient’s willingness to receive tuberculosis treatment management (%)

Categories	Willing	Perfect means of management				Unwilling	Reasons for unwilling			
		Telephone	Message	Home visit	Other means		Social stigma	Unnecessary	Inconvenient	Other reasons
Management in intensive phase (*n* = 637)	387 (60.80)	347 (89.7)	9 (2.3)	105 (27.1)	1 (0.3)	250 (39.2)	115 (46.0)	116 (46.4)	23 (9.2)	1 (0.4)
Management in continuation phase (*n* = 636)	387 (60.8)	349 (90.2)	8 (2.1)	93 (24.0)	1 (0.3)	249 (39.2)	113 (45.4)	118 (47.4)	21 (8.4)	1 (0.4)
Remind of follow-up (*n* = 620)	562 (90.6)	535 (95.2)	6 (1.1)	84 (14.9)	1 (0.2)	58 (9.4)	15 (25.9)	25 (43.1)	4 (6.9)	10 (17.2)
Management on missed dose (*n* = 612)	547 (89.4)	501 (91.6)	16 (2.9)	104 (19.0)	10 (1.8)	65 (10.6)	15 (23.1)	38 (58.5)	5 (7.7)	6 (9.2)
Management on interrupted treatment (*n* = 617)	554 (89.8)	501 (90.4)	15 (2.7)	111 (20.0)	10 (1.8)	63 (10.2)	14 (22.2)	41 (65.1)	5 (7.9)	2 (3.2)

**Table 3:** Multivariate analysis for factors associated with patient’s self-administrated tuberculosis treatment

Variable	Self-administrated TB treatment in whole period (95% *CI*)	Self-administrated TB treatment in intensive phase (95% *CI*)	Self-administrated TB treatment in continuation phase (95% *CI*)
Age			
< 20	Reference	Reference	Reference
20–40	0.80(0.33–1.94)	0.70 (0.29–1.67)	0.81 (0.42–1.54)
40–60	0.90(0.34–2.39)	0.75 (0.28–1.97)	1.05 (0.54–2.06)
≥ 60	0.52(0.18–1.51)	0.44 (0.15–1.26)	0.70 (0.35–1.41)
Ethnicity			
Han Race	Reference	Reference	Reference
Others	3.35(1.96–5.73)	3.13 (1.81–5.40)	3.52 (2.02–6.13)
Residence			
Urban	Reference	Reference	Reference
Rural	2.34(1.06–5.13)	2.19 (1.01–4.74)	1.42 (0.74–2.73)
Registered information			
Resident	Reference	Reference	–
Migrant	0.41(0.20–0.84)	0.51 (0.25–1.03)	–
Education			
Primary and below	Reference	Reference	–
Junior middle school	0.54(0.30–0.97)	0.59 (0.33–1.06)	–
High school and above	1.06(0.48–2.36)	0.92 (0.42–2.04)	–
Occupation			
Staff/Cadre/Retiree	Reference	Reference	–
Self-employed	1.21(0.34–4.27)	1.05 (0.30–3.68)	–
Farmer/Migrant worker	1.28(0.44–3.75)	1.31 (0.44–3.84)	–
Student	0.68(0.18–2.52)	0.73 (0.20–2.71)	–
Others	1.97(0.61–6.36)	1.82 (0.57–5.88)	–
TB burden			
Low	Reference	Reference	Reference
Middle	0.17(0.09–0.31)	0.14 (0.08–0.27)	0.23 (0.13–0.42)
High	0.25(0.14–0.45)	0.24 (0.13–0.44)	0.40 (0.23–0.73)
AFB smear status			
Negative	Reference	Reference	–
Positive	1.12(0.72–1.75)	1.17 (0.75–1.82)	–
First health facility for consultation			
Primary health facility	Reference	Reference	Reference
Non-primary health facility	1.73(0.96–3.11)	1.71 (0.96–3.04)	1.50 (0.88–256)
Nearest health institution			
Primary health facility	Reference	Reference	Reference
Non-primary health facility	1.89(1.09–3.28)	1.80 (1.04–3.11)	1.89 (1.11–3.22)
Willingness to TB treatment management			
Full acceptance	Reference	Reference	Reference
Non-acceptance	6.79(4.13–11.16)	6.35 (3.85–10.48)	5.18 (3.20–8.38)

*TB* Tuberculosis, *CI* Confidence interval, *AFB* Acid-fast bacilli

### Satisfaction of TPM by LHWs

**Fig. 3:**
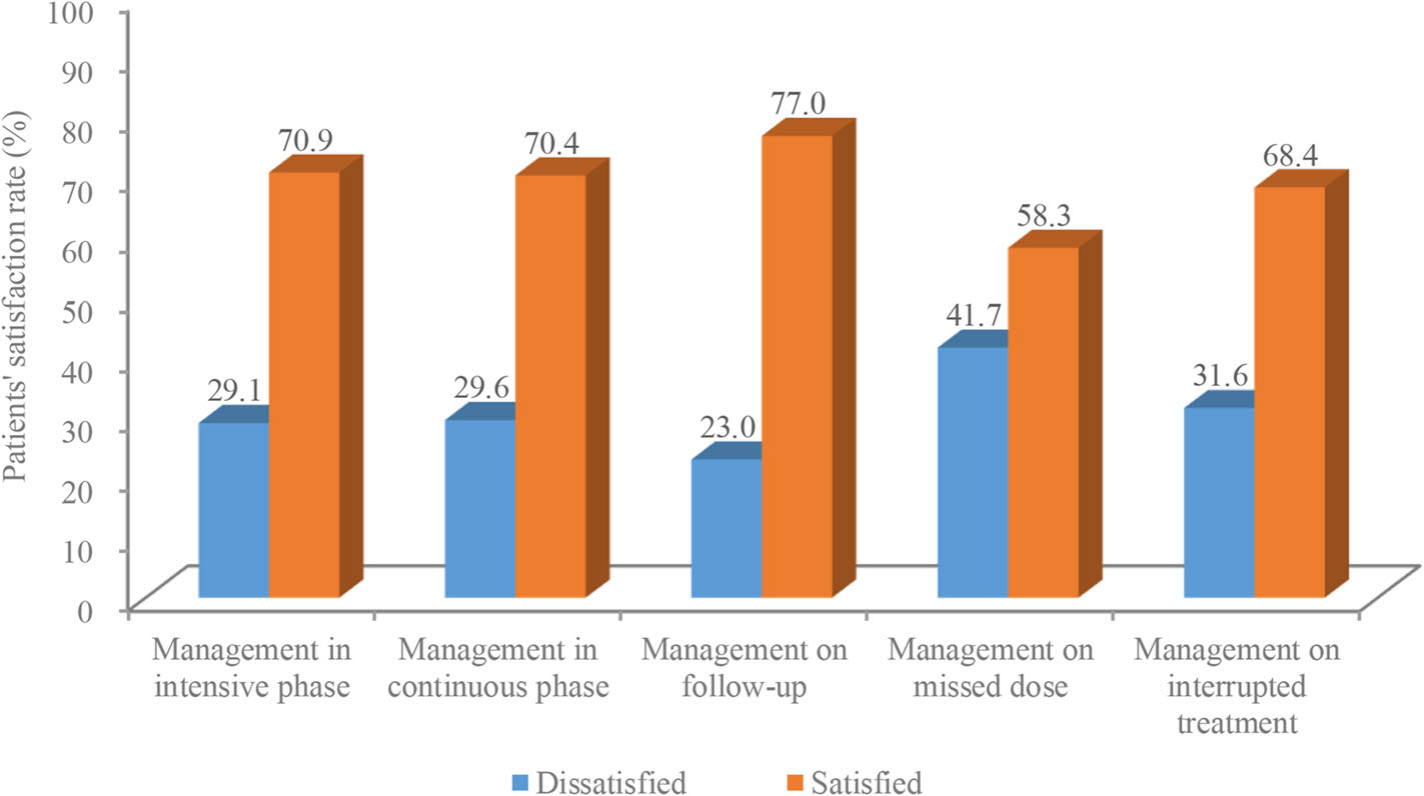
Tuberculosis patient’s satisfaction to management by lay health workers in primary health care sectors in Guizhou Province (%). This figure demonstrated the percentage of tuberculosis patient satisfied/dissatisfied to management in intensive phase–continuous phase–missed dose–interrupted treatment–and sputum follow-up

Among TB patients who received TPM, 70.9% (*n* = 161) and 70.4% (*n* = 143) of patients were generally satisfied with TPM by LHWs during the intensive and continuation treatment phase, respectively. Regarding the remainder in each specific situation of noncompliance, 7.0% showed satisfaction with reminders for sputum follow-up. Additionally, 68.4% of TB patients who had missed doses and 58.3% of TB patients who experienced interrupted treatment were satisfied with TPM by LHWs (Fig. 3).

### Factors associated with TPM

The results of the *χ*^2^ test indicated that age, ethnicity, residence, TB prevalence, initiated institute for treatment, medical institute close to patients’ home and patients’ willingness to accept being supervised were potentially associated with self-administered drug intake during the entire treatment period as well as the intensive phase and continuation phase of treatment separately. In addition, migrants, educational level, occupation and type of TB patients by sputum smear test were associated with self-administered treatment during the entire treatment period and the intensive phase (*P* < 0.05) (see Additional file 3).

Multivariate logistic regression analysis of factors associated with self-administered treatment indicated that being ethnic minorities, living in a rural area, the nearest health institution being non-PHC, and non-acceptance of TB treatment would significantly increase the possibility of conducting self-administered treatment during the entire treatment period (*P* < 0.05), and being ethnic minorities (*OR* = 3.35, 95% *CI*: 1.96–5.73) and non-acceptance of TB treatment (*OR* = 6.79, 95% *CI*: 4.13–11.16) are the two strongest effectors. Meanwhile, being a migrant, with junior school education and living in an area with middle and high TB burden are associated with a higher likelihood of receiving TPM (*P* < 0.05), especially for the middle (*OR =* 0.17, 95% *CI*: 0.09–0.31) and high (*OR* = 0.25, 95% *CI*: 0.14–0.45) burden area compared to the low burden area. Regarding the analysis of intensive and continuation phases separately, being ethnic minorities, the nearest health institution being non-PHC and non-acceptance of TB treatment are still significantly correlated with a higher likelihood of self-administered treatment in both phases while the impact of living in a rural area is only statistically significant for the intensive phase. The association between living in middle and high TB burden areas and a higher possibility of receiving TPM is still statistically significant in both phases, but the effects of being a migrant and having junior middle school education for the two separate phases are not significant (Table 3).

## Discussion

This study showed that more than half of the patients self-administered treatment in both the intensive phase and continuation phase in Guizhou Province even under the integrated TB control model. While the study reveals a higher TPM management rate compared to the result from one study in Chongqing, a similar area regarding economic development where only 16% of TB patients were supervised when taking anti-TB drugs in CDC model [6], the study also shows no improvement compared with the finding from one previous meta-analysis [5].

This study also revealed that TB patients who lived in rural areas, far away from PHC sectors and who were unwilling to receive TPM because of social stigma or no perceived need were more likely to experience self-administered treatment under the integrated model. These results were consistent with previous studies under the CDC model where more rural patients tended to self-administer treatment due to poverty, mobility, low education level, TB-related social stigma, lack of health consciousness, etc. [6, 10–13], implying that these factors continue to impede the implementation of TPM under the new model. In addition, our study further discovered that patients of ethnic minorities were more likely to conduct self-administered treatment, potentially due to culture differences or other unknown issues that are worth further exploration. As Guizhou and other parts of western China have large populations of multiple ethnic minorities, future research on the TPM of ethnic minorities is critical to inform policies aimed at improving overall TPM coverage [23]. Notably, this research also demonstrated that TB patients from counties with higher TB burden were supervised better than counties with low TB burden, most likely because TPM was emphasized more in those regions.

As the unwillingness to accept TPM remains the major challenge to improve TPM, recent research also explored how TPM could be better designed and tailored to local patients’ needs. Community-based DOT (CB-DOT), designed to relieve the pressure of patient care on overstretched health facilities in countries with a high TB burden [24], is a cost-effective approach associated with better compliance to treatment and better patient satisfaction compared to hospital-based DOT (HB-DOT) [25]. Systematic review [26–28] also showed that CB-DOT did improve TB treatment outcomes. In China, TPM by LHWs in the integrated TB control model [6] is one type of CB-DOT. Studies have also found that “patient-centered” and “community-centered” DOT did improve treatment outcomes if tailored to local community conditions [26], including the choice of supervisors. Future research may focus on how to improve the design of TPM to be more patient-centered under the integrated TB model, ensuring patient’s confidentiality and acceptability of supervised treatment simultaneously.

The acceptability of TPM may also be improved through an innovative care delivery approach with the help of modern technology. This study found that the preferred TPM approach is phone reminder, rather than home visit, which is similar to previous reports in other regions [29–33]. Zhang et al. also reported the feasibility of management on TB patients through WeChat with a promising prospect [32]. This approach deserves more research, as it has the potential to protect TB patients from social stigma and improve the feasibility of TPM delivery, particularly in remote mountain areas with transportation difficulties. Digital technologies have been recommended as approaches for TPM by WHO and the 13th Five year national TB program in China [34, 35]. With the advance of e-health development, new technologies should be included in TPM once evidence confirms its effectiveness in improving TPM.

This study also has several limitations. It is difficult to explain our finding that the migrant patients in our sample were better supervised, and this is perhaps due to the small proportion of migrants in our sample, which restricts our exploration into the actual correlation between migrants and TPM. Additionally, this study assessed TPM and identified needs of TPM only by structured questionnaires with TB patients, thus the expression of patient needs is limited within the predetermined structured questions. To develop a patient-centered, culturally sensitive TPM intervention for TB control, a participatory study (by qualitative research methods, such as focus group discussion) with both TB patients and LHWs in PHC sectors to elicit patients’ perceived needs of TPM and, causes of non-acceptance to TPM, and to identify the possible approach to improve TPM delivered by a local community is necessary. In future studies, mixed research methods can be used to collect data from TB patients, health service providers (TB doctors and LHWs in PHC sectors) and health service buyers (e.g., related policy makers).

## Conclusions

TPM by LHWs was not well-delivered according to the TB national program in West China though it was emphasized in the new integrated TB model in China, as indicated by the low coverage and quality of TPM in the intensive and continuous phase of TB treatment. The impeding factors of TPM, such as non-acceptance to TPM, ethnic minorities, and being far from PHC, need to be further addressed to increase TPM coverage. Strengthening patient-centered, community-based TPM for TB patients could be a possible approach, and locally appropriate measures should be taken to identify and address physical, financial, social and cultural (as well as health system) obstacles to access patient-centered, community-based TPM. This study has global significance in providing evidence to improve TPM in resource-limited regions through the effective involvement of LHWs.

## Additional files

Additional file 1: Multilingual abstracts in the five official working languages of the United Nations. (PDF 578 kb)

Additional file 2: Means of TB treatment management by LHWs in Guizhou province. (DOCX 16 kb)

Additional file 3: Univariate analysis factors associated with patient’s self-administered TB treatment. (DOCX 22 kb)

## Data Availability

Data sharing is not applicable to this article as no datasets were generated or analysed during the study.

## Abbreviations

AIDS: Acquired immunodeficiency syndrome
CB-DOT: Community-based directly observed treatment
CDC: Centers for Disease Control and Prevention
CHCs: Community health centers
*CI*: Confidence interval
DOT: Directly observed treatment
DOTs: Directly observed treatment, short-course
HBDOT: Hospital-based directly observed treatment
HIV: Human immunodeficiency virus
LHWs: Lay health workers
MDR-TB: Multidrug-resistant tuberculosis
NTP: National tuberculosis control program
*OR*: Odds ratio
PHC: Primary health care
PTB: Pulmonary tuberculosis
RR-TB: Rifampicin-resistant tuberculosis
TB: Tuberculosis
THCs: Township health centers
TPM: TB patient management
WHO: World Health Organization
