# Supplementary material for: Is tuberculosis patients management improved in the integrated TB control model in West China? A survey in Guizhou Province, China
Source: Infect Dis Poverty. 2019 Jul 2;8:55. doi: 10.1186/s40249-019-0563-3 (PMC6604227; doi:10.1186/s40249-019-0563-3)
Supplement: Supplementary file 1 — Multilingual abstracts in the five official working languages of the United Nations. (PDF 578 kb) [file 40249_2019_563_MOESM1_ESM.pdf]

Translation of the abstract into the five official working languages of the United Nations

؟ الصين غرب في السل لمكافحه المتكامل النموذج في السل مرضي أداره تحسنت هل

Jie Pu, Wei Chen, Wei-Xi Jiang, Wei Xing, Sheng-Xiang Liang, Geng Wang, Shi- Li Liu, Hao Wu, Ying Li, Sheng-Lan Tang

مجرده

مراكز أو بالسل صابين‌الم المرضي أداره مستوصفات قدمتها التي التغطية تكن ولم .للعلاج المريض امتثال لتحسين الاهميه بالغ أمر السل مرضي أداره **:الخلفية**
لمكافحه كامل‌المت النموذج وفي .الصين في السل بمكافحه الخاص الامراض لمكافحه السابقة المراكز نموذج اطار في مرتفعه منها والوقاية الامراض مكافحه
إلى الدراسة هذه تهدف .رئيسي بشكل السل مرضي أداره بتوفير الاوليه الصحية الرعاية قطاعات في العامة الصحة مجال في العاملون قام ، الصين في السل
الصحيين العاملين بلة‌ من السل لمرض المرضي تنفيذ علي تؤثر التي العوامل وتحديد الموارد المحدودة الصين غرب في السل مرضي أداره تنفيذ في التحقيق
.توبيركولوسي المتكامل التحكم نموذج اطار في .العلمانيين
قويتشو مقاطعه في مختارة‌اال المقاطعات/المقاطعات من سنه 15 ≥ بولموناريتوبيركولوسيباتينتس .الدراسة مواقع لاختيار طبقيا شوائيةء عينات استخدمت :الطرق
المرتبطة العوامل لتحديد اللوجستي والانحدار χ استخدمت وقد .البيانات لجمع منظمه استبيانات واستخدمت .2016 مايو إلى 2015 أغسطس من مسحها تم
.(بالسل المصابين غير المرضي أداره) الذاتية بالمعالجة
وكان .العرقية الأقليات من المرضي من المائة في 30 قرابة وكان .النهائي التحليل في بولموناريتوبيركولوسي المرضي تضمين تم 638 ، المجموع في **:النتائج**
من 37.1% فقط .للعلاج الامتثال سوء من يعانون الذين المرضي من ٪ 24.9 و ، توبيركولوسي عبء ارتفاع مع المقاطعات من المرضي من ٪ 30 من أكثر
وشملت .العلاج فتره طوال توبيركولوسي المتكامل التحكم نموذج تحت العلمانيين الصحيين العاملين قبل من الاداره قبل من السل مرضي أداره تلقوا المرضي
كان (3.35 = أو) اثنيه أقليات وكونها .متصوره حاجه وجود وعدم الاجتماعية العار وصمه عنهم ابلغ الذين المرضي أداره في الرغبة لعدم الرئيسية الأسباب
تلقي احتمال من ديزي‌ قد السل نم‌ مرتفع أو متوسط عبء ذات مناطق في العيش ان حين في ، السل مرضي أداره تلقي احتمال بانخفاض مرتبطا رئيسيا عاملا
لأنهم فـبالهاة‌ تذكيرا المرضي من المائة في 85 من أكثر اختار ، الحالية الاداريه النهج بين ومن .(التوالي علي ، 0.25 و 0.17 = OR) أداره السل مريض
.العلمانيين الصحيين العاملين قبل من السل مرضي أداره يفضلون
المعيقة العوامل جه‌معاال وينبغي ، التحسين من مزيد إلى وتحتاج منخفضه الصين غرب في المتكامل النموذج اطار في السل مرضي ه‌أدار تزال لا **:الاستنتاجات**
البحوث في بالسل ي‌المرضـ لتسليم جدوى أكثر نهج ووضع والمجتمعية المريض علي المرتكزة السل مرضي أداره تعزيز استكشاف وينبغي .السل مرضي لأداره
.المنطقة هذه في لة‌االمقب

Translated from English version into Arabic by Hamza Shaffison, through

中山大學医学院
SUN YAT-SEN UNIVERSITY SCHOOL OF MEDICINE

中国西部地区结核病综合防治模式下结核病患者管理情况是否有所改善？
-----贵州省调查研究结果

蒲傑，陈玮，江蔚曦，邢伟，梁胜翔，王赓，刘诗莉，吴昊，李颖，汤胜蓝

摘要

引言：结核病患者管理（TPM）对提高患者治疗依从性至关重要。在中国以往的以疾控中心（CDC）为主的结核病防治模式下，由结防所或疾控中心提供结核病患者管理，其覆盖率不高。在综合防治模式下，主要通过基层医疗卫生服务机构的医务人员对结核病患者进行管理。本研究旨在对我国资源匮乏的西部地区综合防治模式下社区结核病管理现状及影响因素进行探索。

方法：利用分层随机抽样方法选取研究地点。在 2015 年 8 月至 2016 年 5 月期间，利用问卷对 15 周岁及以上的肺结核患者进行调查，采用卡方检验和二分类 logistic 回归分析方法分析患者管理的相关因素。

结果：共有 638 名肺结核患者纳入了最终的分析，其中近 30%的患者为少数民族，超过 30%的患者来自 TB 高疫

情地区，24.9%的患者治疗依从性较差。仅有 37.1%的患者在治疗期间接受过基层医务人员的督导管理。患者不愿意接受督导管理的主要原因包括社会歧视和认为没有必要被管理。少数民族患者在治疗期间更难接受管理（*OR*=3.35），而疫情中等或高发地区的患者更容易接受治疗管理（疫情中等地区 *OR*=0.17，疫情高发地区 *OR*=0.25）。在现有的管理方式中，超过 85%的患者首选基层医务人员电话督导。

结论：中国西部地区综合防治模式下结核病患者管理率有待进一步提高，且需要针对结核病患者管理相关因素采取措施。下一步需要针对加强以病人为中心和以社区为基础的结核病患者管理模式和创新患者管理方式进行深入研究。

Translated from English version into Chinese by Jie Pu

Le suivi des patients atteints de la tuberculose est-il plus efficace avec le programme antituberculeux intégré en Chine occidentale?

Jie Pu, Wei Chen, Wei-Xi Jiang, Wei Xing, Sheng-Xiang Liang, Geng Wang, Shi- Li Liu, Hao Wu, Ying Li, Sheng-Lan Tang

Résumé

Contexte: Le suivi des patients tuberculeux (TPM, en anglais) est crucial pour parvenir à améliorer l’observance du traitement. Le TPM offert par les dispensaires antituberculeux ou par les centres de lutte et de prévention était médiocre sous le modèle antérieur chinois de lutte contre la tuberculose. Sous le programme chinois antituberculeux intégré, il a été noté que le TPM était principalement assuré par du personnel soignant non professionnel dans les secteurs de soins de santé primaires. La présente étude examine le TPM dans les régions à ressources limitées de la Chine occidentale, en vue d’identifier les facteurs freinant sa distribution par le personnel soignant non-professionnel sous le programme antituberculeux intégré.

Méthodes: Nous avons utilisé une méthode d’échantillonnage stratifiée pour sélectionner les zones de notre étude. L'étude, menée entre août 2015 et mai 2016, a porté sur des patients âgés de 15 ans ou plus atteints de tuberculose pulmonaire (TP) et vivant dans certains districts ou cironscriptions de la province de Guizhou. Des questionnaires structurés ont été utilisés afin de recueillir les données. Des méthodes d'analyse, telles que le test χ^2 et la régression logistique ont été utilisées pour identifier les facteurs associés aux traitements auto-administrés (hors TPM).

Résultats: Au total, 638 patients atteints de TP ont été inclus dans l’étude finale. Près de 30 % de ces patients appartenaient à des minorités ethniques. Plus de 30 % d'entre eux provenaient de circonscriptions très touchées par la tuberculose, et 24,9 % ne suivaient pas correctement leur traitement. Seuls 37,1 % des patients ont bénéficié du TPM réalisé par le personnel soignant non professionnel dans le cadre du programme antituberculeux intégré sur toute la période du traitement. Les principales raisons rapportées par les patients pour expliquer cette réticence vis-à-vis du suivi comprenaient la stigmatisation sociale et l’absence de réelle perception d'un besoin de prise en charge. L’appartenance à une minorité ethnique (*OR* = 3,35) s'est avérée un facteur majeur associé à une plus faible probabilité de bénéficier du TPM, tandis que les patients vivant dans des zones moyennement ou fortement touchées par la tuberculose étaient plus susceptibles de bénéficier de ce suivi (*OR* = 0,17 et 0,25, respectivement). Aujourd'hui, parmi les méthodes de gestion et de suivi du traitement, plus de 85 % des patients ont choisi les rappels par téléphone (effectués par le personnel soignant non-professionel).

Conclusions: Le TPM, dans le cadre du programme intégré en Chine occidentale, reste insuffisant, doit être amélioré, et les facteurs qui lui font obstacle doivent être pris en considération. Lors de futures études dans la région, nous recommandons de recentrer le TPM sur les besoins du patient, de le renforcer sur le plan communautaire, et d’élaborer des approches plus facilement réalisables pour sa distribution.

Translated from English version into French by Suzanne Assenat, proofread by Delphine Tomlins, through

Улучшилась ли работа с пациентами при интегрированной модели контроля туберкулеза на западе Китая?

Цзиэ Пу, Вэй-Си Цзянь, Вэй Син, Шэнь-Сянь Лянь, Гэн Ван, Ши-Ли Лю, Хао У, Инь Ли, Шэнь-Лань Тан

Реферат

Предпосылки: работа с туберкулезными (ТБ) пациентами (РТП) крайне важна для улучшения готовности пациента к лечению. При предыдущей модели контроля ТБ в Китае на базе центров контроля и профилактики заболеваемости (ЦКЗ) в ТБ-диспансерах или в самих ЦКЗ в РТП участвовала лишь невысокая доля пациентов. В интегрированной модели контроля ТБ в Китае работа с ТБ пациентами осуществлялась в основном работниками здравоохранения низового уровня (LHW) в секторах первичной медико-санитарная помощи (ПМСП). В настоящей работе исследуется постановка РТП в бедном ресурсами западном Китае и то, насколько эффективностьРТП зависит от работы LHW при интегрированной модели контроля ТБ.

Методы: для выбора мест для исследования применялась стратифицированная случайная выборка**.** Пульмонологические пациенты с ТБ (ПТБ) старше 15 лет из отдельных выбранных уездов/районов провинции Гуйчжоу наблюдались с августа 2015 г. по май 2016 г. Для сбора данных использовались структурированные вопросники. Для выявления факторов, связанных с самостоятельным лечением (вне РТП) использовались χ^2-тест и логистическая регрессия.

Результаты: всего в окончательном анализе использовались данные 638 ПТБ-пациентов. Около 30 % пациентов были представителями этнических меньшинств. Более 30 % пациентов было из уездов с высокой ТБ-нагрузкой, а 24,9 % пациентов плохо относились к лечению. РТП посредством LHW в рамках интегрированной модели контроля ТБ в течении всего периода лечения было охвачено лишь 37,1 % пациентов. Основными причинами нежелания участвовать в РТП пациенты называли социальную стигму и отсутствие чувства потребности. Принадлежность к этническим меньшинствам (*отношение шансов* = 3,35) являлась основным фактором, связанным с низкой вероятностью участия в РТП, а проживание в зонах со средней или с высокой ТБ-нагрузкой соотносилось с повышенным участием в РТП (*отношение шансов* = 0,17 и 0,25, соответственно). При существующей организации работы более 85 % пациентов в качестве предпочтительной РТП выбирало телефонное напоминание со стороны LHW.

Выводы: РТП при интегрированной модели на западе Китая все еще находится на низком уровне и нуждается в дальнейшем совершенствовании; необходимо уделить внимание факторам, мешающим РТП. В будущем при исследовании данного региона необходимо уделять внимание укреплению РТП, в центре которой находится пациент, и работе с этническими сообществами, выработав более гибкий подход к осуществлению РТП.

Translated from English version into Russian by Alexander Somin, proofread by Michael Orlov, through

¿Ha mejorado la gestión de pacientes con tuberculosis en el modelo integrado para el control de la tuberculosis en China Occidental?

Jie Pu, Wei Chen, Wei-Xi Jiang, Wei Xing, Sheng-Xiang Liang, Geng Wang, Shi- Li Liu, Hao Wu, Ying Li, Sheng-Lan Tang

Resumen

Antecedentes: la gestión de pacientes con tuberculosis (GPT) es fundamental para mejorar el cumplimiento del tratamiento por parte de estos. La cobertura de la GPT efectuada por dispensarios antituberculosos o por los Centros para el Control y la Prevención de Enfermedades (CCPEEU) no fue alta en el marco del modelo anterior de los CCPEEU para el control de la TB en China. En el modelo integrado para el control de la TB en China, la gestión de pacientes con TB (GPT) fue efectuada principalmente por trabajadores sanitarios legos (TSL) en los sectores de atención primaria de salud (APS). El presente estudio tiene por objeto investigar la ejecución de la GPT en China Occidental, que cuenta con recursos limitados, e identificar los factores que afectan la ejecución de la GPT por parte de los TSL en el marco del modelo integrado para el control de la TB.

Métodos: se utilizó un muestreo aleatorio estratificado para seleccionar los sitios de estudio. Se encuestaron a pacientes con TB pulmonar (TBP) de 15 años o más de los municipios / distritos seleccionados de la provincia de Guizhou entre agosto de 2015 y mayo de 2016. Se utilizaron cuestionarios estructurados para recopilar los datos. Se realizaron una prueba χ^2 y una regresión logística para identificar los factores asociados con el tratamiento autoadministrado (no GPT).

Resultados: en total, se incluyeron 638 pacientes con TBP en el análisis final. Casi el 30% de los pacientes formaban parte de minorías étnicas. Más del 30% de los pacientes provenía de municipios con alta incidencia de TB, y el 24,9% de los pacientes presentaba un cumplimiento deficiente del tratamiento. Solo el 37,1% de los pacientes recibió una GPT efecuatada por TSL en el marco del modelo integrado para el control de la TB a lo largo del período de tratamiento. Las razones principales de la renuencia a la gestión indicadas por los pacientes incluyeron el estigma social y la no percepción de una necesidad. El hecho de ser minorías étnicas (*OR* = 3,35) fue un factor importante asociado con la menor probabilidad de recibir una GPT, mientras que vivir en zonas con una incidencia media o alta de TB puede aumentar la probabilidad de recibir una GPT (*OR* = 0,17 y 0,25, respectivamente). De entre los enfoques de gestión actuales, más del 85% de los pacientes eligió un recordatorio telefónico como su GPT preferida por parte de los TSL.

Conclusiones: la GPT en el marco del modelo integrado en China Occidental sigue siendo baja y debe mejorarse aún más; asimismo, deben abordarse los factores que impiden la GPT. En futuras investigaciones en esta región, debe explorarse el fortalecimiento de la GPT centrada en los pacientes y basada en la comunidad, así como el desarrollo de enfoques más factibles en cuanto a la realización de la GPT.

Translated from English version into Spanish by Mayra León, proofread by Maria Gracia Zavarse, through

TRANSLATORS
WITHOUT BORDERS
