# Supplementary material for: Is tuberculosis patients management improved in the integrated TB control model in West China? A survey in Guizhou Province, China
Source: Infect Dis Poverty. 2019 Jul 2;8:55. doi: 10.1186/s40249-019-0563-3 (PMC6604227; doi:10.1186/s40249-019-0563-3)
Supplement: Supplementary file 2 — Means of TB treatment management by LHWs in Guizhou province. (DOCX 16 kb) [file 40249_2019_563_MOESM2_ESM.docx]

**Additional file 2 Means of TB treatment management by LHWs in Guizhou province n (%)**

| **Categories** | **Management means** | | | |
| --- | --- | --- | --- | --- |
|  | **Telephone** | **Short message** | **Home visit** | **Other means** |
| **Medication management (n = 237)** | 223  (94.1) | 6  (2.5) | 127  (53.6) | 13  (5.5) |
| **Remind of follow-up**  **(n = 363)** | 340  (93.7) | 28  (7.7) | 96  (26.4) | 31  (8.5) |
| **Management on missed dose (n = 31)** | 31  (100) | 9  (29.0) | 8  (25.8) | 8  (25.8) |
| **Management on interrupted treatment**  **(n = 25)** | 21  (84.0) | 0  (0) | 12  (48.0) | 0  (0) |
