# Supplementary material for: Is tuberculosis patients management improved in the integrated TB control model in West China? A survey in Guizhou Province, China
Source: Infect Dis Poverty. 2019 Jul 2;8:55. doi: 10.1186/s40249-019-0563-3 (PMC6604227; doi:10.1186/s40249-019-0563-3)
Supplement: Supplementary file 3 — Univariate analysis factors associated with patient’s self-administered TB treatment. (DOCX 22 kb) [file 40249_2019_563_MOESM3_ESM.docx]

**Additional file 3 Univariate analysis factors associated with patient’s self-administrated TB treatment**

| **Variable** | **Self-administrated TB treatment in whole period** | **P (χ² test)** | **Self-administrated TB treatment in intensive phase** | **P (χ² test)** | **Self-administrated TB treatment in continuation phase** | **P (χ² test)** |
| --- | --- | --- | --- | --- | --- | --- |
| **Age** |  |  |  |  |  |  |
| < 20 | 39(52.0) | < 0.05 | 42 (56.0) | < 0.05 | 48 (64.0) | < 0.05 |
| 20-40 | 125(54.6) |  | 129 (56.3) |  | 137 (59.8) |  |
| 40-60 | 126(64.9) |  | 129 (66.5) |  | 138 (71.1) |  |
| ≥ 60 | 68(48.6) |  | 70 (50.0) |  | 76 (54.3) |  |
| **Gender** |  |  |  |  |  |  |
| Male | 220(56.6) | 0.75 | 228 (58.6) | 0.66 | 248 (63.8) | 0.41 |
| Female | 137(55.2) |  | 141 (56.9) |  | 150 (60.5) |  |
| **Ethnicity** |  |  |  |  |  |  |
| Han Race | 198(44.2) | < 0.05 | 208 (46.4) | < 0.05 | 233 (52.0) | < 0.05 |
| Others | 157(84.9) |  | 159 (85.9) |  | 163 (88.1) |  |
| **Residence** |  |  |  |  |  |  |
| Urban | 22(33.8) | < 0.05 | 23 (35.4) | < 0.05 | 31 (47.7) | < 0.05 |
| Rural | 336(58.7) |  | 347 (60.7) |  | 368 (64.3) |  |
| **Registered information** |  |  |  |  |  |  |
| Resident | 328(58.3) | < 0.05 | 337 (59.9) | < 0.05 | 356 (63.2) | 0.28 |
| Migrant | 27(39.1) |  | 30 (43.5) |  | 39 (56.5) |  |
| **Marital status** |  |  |  |  |  |  |
| Single | 82(53.2) | 0.249 | 86 (55.8) | 0.19 | 94 (61.0) | 0.10 |
| Married | 249(58.8) |  | 257 (60.3) |  | 277 (65.0) |  |
| Devoiced/Widowed | 24(48.0) |  | 24 (48.0) |  | 25 (50.0) |  |
| **Education** |  |  |  |  |  |  |
| **Variable** | **Self-administrated TB treatment in whole period** | **P (χ² test)** | **Self-administrated TB treatment in intensive phase** | **P (χ² test)** | **Self-administrated TB treatment in continuation phase** | **P (χ² test)** |
| Primary and below | 188(60.6) | < 0.05 | 193 (62.3) | < 0.05 | 201 (64.8) | 0.22 |
| Junior middle school | 81(45.8) |  | 87 (49.2) |  | 101 (57.1) |  |
| High school and above | 86(58.9) |  | 87 (59.6) |  | 93 (63.7) |  |
| **Occupation** |  |  |  |  |  |  |
| Staff/Cadre/Retiree | 21(50.0) | < 0.05 | 21 (50.0) | < 0.05 | 23 (54.8) | 0.49 |
| Self-employed | 14(42.4) |  | 14 (42.4) |  | 17 (51.5) |  |
| Farmer/Migrant worker | 267(59.6) |  | 276 (61.6) |  | 288 (64.3) |  |
| Student | 32(57.1) |  | 34 (60.7) |  | 35 (62.5) |  |
| Others | 24(40.7) |  | 25 (42.4) |  | 36 (61.0) |  |
| **Main source of income** |  |  |  |  |  |  |
| Patients | 61(56.5) | 0.49 | 62 (57.4) | 0.57 | 66 (61.1) | 0.56 |
| Others | 131(53.5) |  | 137 (55.9) |  | 149 (60.8) |  |
| Shared with other | 163(58.6) |  | 168 (60.4) |  | 181 (65.1) |  |
| **Economic status** |  |  |  |  |  |  |
| Labor force | 238(57.3) | 0.50 | 243 (58.6) | 0.84 | 266 (64.1) | 0.37 |
| Dependent | 120(54.5) |  | 127 (57.7) |  | 133 (60.5) |  |
| **Type of health insurance** |  |  |  |  |  |  |
| Basic health insurance | 348(56.8) | 0.21 | 360 (58.7) | 0.17 | 385 (62.8) | 0.85 |
| Others | 10(43.5) |  | 10 (43.5) |  | 14 (60.9) |  |
| **TB burden** |  |  |  |  |  |  |
| Low | 155(85.6) | < 0.05 | 159 (87.8) | < 0.05 | 158 (87.3) | < 0.05 |
| Middle | 56(25.3) |  | 59 (26.7) |  | 76 (34.4) |  |
| High | 147(62.3) |  | 152 (64.4) |  | 165 (69.9) |  |
| **Variable** | **Self-administrated TB treatment in whole period** | **P (χ² test)** | **Self-administrated TB treatment in intensive phase** | **P (χ² test)** | **Self-administrated TB treatment in continuation phase** | **P (χ² test)** |
| **Type of patient** |  |  |  |  |  |  |
| New | 317(56.6) | 0.50 | 328 (58.6) | 0.43 | 351 (62.7) | 0.85 |
| Retreatment | 41(52.6) |  | 42 (53.8) |  | 48 (61.5) |  |
| **AFB smear status** |  |  |  |  |  |  |
| Negative | 229(59.9) | < 0.05 | 236 (61.8) | < 0.05 | 249 (65.2) | 0.09 |
| Positive | 129(50.4) |  | 134 (52.3) |  | 150 (58.6) |  |
| **First health facility for consultation** |  |  |  |  |  |  |
| Primary health facility | 30(31.3) | < 0.05 | 32 (33.3) | < 0.05 | 38 (39.6) | < 0.05 |
| Non-primary health facility | 328(60.5) |  | 338 (62.4) |  | 361 (66.6) |  |
| **Nearest health institution** |  |  |  |  |  |  |
| Primary health facility | 281(54.5) | 0.07 | 291 (56.4) | < 0.05 | 311 (60.3) | < 0.05 |
| Non-primary health facility | 7(63.6) |  | 79 (65.3) |  | 88 (72.7) |  |
| **Willingness to TB treatment management** |  |  |  |  |  |  |
| Full acceptance | 142(37.4) | < 0.05 | 151 (39.7) | < 0.05 | 174 (45.8) | < 0.05 |
| Non-acceptance | 215(83.7) |  | 218 (84.8) |  | 224 (87.2) |  |
